# 512. Does Time From COVID-19 Symptom Onset to Administration of Anti-spike Protein Monoclonal Antibody Predict Response?

**DOI:** 10.1093/ofid/ofab466.711

**Published:** 2021-12-04

**Authors:** Savanna SanFilippo, Brynna Crovetto, Marc Milano, John Bucek, Ronald G Nahass, Luigi Brunetti

**Affiliations:** 1 RWJBarnabas Health, Somerville, New Jersey; 2 RWJBarnabas Health - RWJ Somerset, Somerville, New Jersey; 3 ID Care, Hillsborough, New Jersey; 4 Rutgers, The State University of New Jersey, Piscataway, New Jersey

## Abstract

**Background:**

Casirivimab/imdevimab is a monoclonal antibody (mAb) cocktail with emergency use authorization for mild-to-moderate coronavirus disease 2019 (Covid-19) in patients at high risk for severe disease progression and/or hospitalization. Little is known about the importance of early administration of this product. The objective of this study was to determine if early administration (within 3 days of symptom onset) of casirivimab/imdevimab is associated with better outcomes.

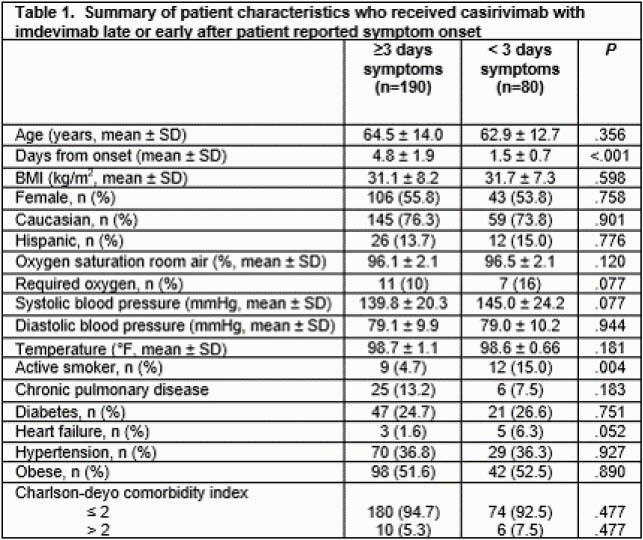

**Methods:**

Single-center, retrospective cohort study including all consecutive patients who received casirivimab/imdevimab at our institution through May 2021. The primary outcome was 30-day post-infusion hospital admission rate in patients who received mAb ≥ 3 days (later) or < 3 days (early) in relation to patient reported symptom onset. Secondary outcomes included any hospital revisit within 30-days. Adverse events were also captured. Chi-square and independent samples t-test were used to compare categorical and continuous data, respectively. Multivariable logistic regression was used to adjust for confounders.

**Results:**

270 patients met the inclusion criteria and were included in the analysis. There were 80 patients with early administration and 190 with later administration. Baseline characteristics for both groups were similar. Mean age was approximately 64 years and BMI 31 mg/m^2^. Table 1 provides a summary of patient characteristics. Late and early administration of casirivimab/imdevimab were similar in terms of hospital admission for any therapy related failure within 30 days of mAb administration after adjusting for age and Charlson comorbidity index (3.7% vs. 7.5%; adjusted odds ratio 0.69, 95% confidence interval, 0.20 – 2.39; p=0.561). Similarly, there were no significant differences in any hospital revisit.

**Conclusion:**

We did not find any difference in outcomes between early and late administration of casirivimab/imdevimab.

**Disclosures:**

**Ronald G. Nahass, MD**, **Abbvie** (Grant/Research Support, Speaker’s Bureau)**Alkermes** (Grant/Research Support)**Gilead** (Grant/Research Support, Speaker's Bureau)**Merck** (Grant/Research Support, Speaker's Bureau)

